# Author Correction: Machine learning assisted quantum super-resolution microscopy

**DOI:** 10.1038/s41467-023-42797-z

**Published:** 2023-10-27

**Authors:** Zhaxylyk A. Kudyshev, Demid Sychev, Zachariah Martin, Omer Yesilyurt, Simeon I. Bogdanov, Xiaohui Xu, Pei-Gang Chen, Alexander V. Kildishev, Alexandra Boltasseva, Vladimir M. Shalaev

**Affiliations:** 1 School of Electrical and Computer Engineering, Birck Nanotechnology Center and Purdue Quantum Science and Engineering Institute, Purdue University, West Lafayette, IN USA; 2 The Quantum Science Center (QSC), a National Quantum Information Science Research Center of the U.S. Department of Energy (DOE), Oak Ridge, TN USA; 3 Department of Electrical and Computer Engineering, University of Illinois at Urbana-Champaign, Urbana, IL USA; 4 Nick Holonyak, Jr. Micro and Nanotechnology Laboratory, University of Illinois at Urbana-Champaign, Urbana, IL USA

**Keywords:** Microscopy, Quantum optics

Correction to: *Nature Communications* 10.1038/s41467-023-40506-4, published online 10 August 2023

The original version of this Article omitted a reference to previous work in ‘Pushkina, A. A., Maltese, G., Costa-Filho, J. I., Patel, P. & Lvovsky, A. I. Superresolution linear optical imaging in the far field. *PRL* **127**, 253602 (2021)’. This has been added as reference 17 at the end of the first sentence of the second paragraph of the Introduction: ‘Another promising route in the realisation of SRM techniques is to take into account the quantum nature of light 13, 14, 15, 16, 17’. This has been corrected in the PDF and HTML versions of the Article.

