# Supplementary material for: Machine learning assisted quantum super-resolution microscopy
Source: Nat Commun. 2023 Aug 10;14:4828. doi: 10.1038/s41467-023-40506-4 (PMC10415374; doi:10.1038/s41467-023-40506-4)
Supplement: Supplementary file 1 — Supplementary Information [file 41467_2023_40506_MOESM1_ESM.pdf]

Supplementary materials
for
"Machine learning assisted quantum super-resolution microscopy"

Zhaxylyk A. Kudyshev[1,2], Demid Sychev[1,2], Zachariah Martin[1,2], Omer Yesilyurt[1,2], Simeon I. Bogdanov[3,4], Xiaohui Xu[1,2], Pei-Gang Chen[1,2], Alexander V. Kildishev[1], Alexandra Boltasseva[1,2], Vladimir M. Shalaev[1,2]

[1] School of Electrical and Computer Engineering, Birck Nanotechnology Center and Purdue Quantum Science and Engineering Institute, Purdue University, West Lafayette, IN, 47907, USA

[2]The Quantum Science Center (QSC), a National Quantum Information Science Research Center of the U.S. Department of Energy (DOE), Oak Ridge, TN 37931, USA

[3]Department of Electrical and Computer Engineering, University of Illinois at Urbana-Champaign, IL 61801, USA

[4] Nick Holonyak, Jr. Micro and Nanotechnology Laboratory, University of Illinois at Urbana-Champaign, IL 61801, USA

1. Convolutional neural network structure and training process. A 1D CNN was used to implement the regression model. Table S1 shows a detailed summary of the regression network structure. Note that an additional input parameter (total number of counts) is concatenated with the output of the feature learning part of the network. This concatenation is performed to introduce additional information into the network to ensure better training outcomes on the HBT datasets with different acquisition times but the same $g^{(2)}(0)$ values.

The CNN model consisted of one input layer, three hidden convolutional layers ("relu" activation function), one max-pooling and dropout layer. The output of the dropout layer is used as an input into the fully connected layer, which consisted of four dense layers and three dropouts. Mean absolute percentage error was used as an error loss function, while the adamax gradient descent optimization was used for training the network. The regression network was trained on 80% of the extended 5s-10s HBT dataset for 100 epochs, while the remaining 20% of the data was used for validation/testing. Figure S1a shows the evolution of the training/validation losses during the training phase.

Layer type	Output shape	Number of parameters	Connected to
Input_1	[215, 1]		
Conv1D_1	[212, 260]	1300	Input_1
Conv1D_2	[209, 260]	270660	Conv1D_1
Conv1D_3	[206, 260]	270660	Conv1D_2
MaxPooling1D	[68, 260]	0	Conv1D_3
Dropout	[68, 260]	0	MaxPooling1D
Input_2	[1]	0	
Flatten	17680	0	Dropout
Concatenate	17681	0	Flatten, Input_2
Dense_1	8041	142180962	Concatenate
Dropout_1	8041	0	Dense_1
Dense_2	4000	32168000	Dropout_1
Dropout_2	4000	0	Dense_2
Dense_3	1000	4001000	Dropout_2
Dropout_3	1000	0	Dense_3
Dense_4	20	20020	Dropout_3
Dense_5	1	21	Dense_4

Table S1. Structure of the CNN regression network.

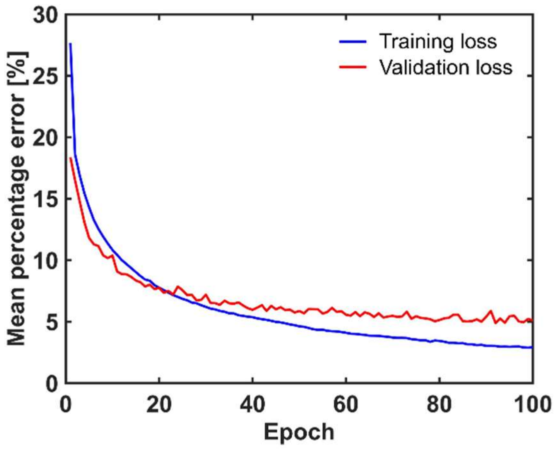

Figure S1. Evolution of the training and validation loss of the CNN regression network during the training.

2. Image formation model.

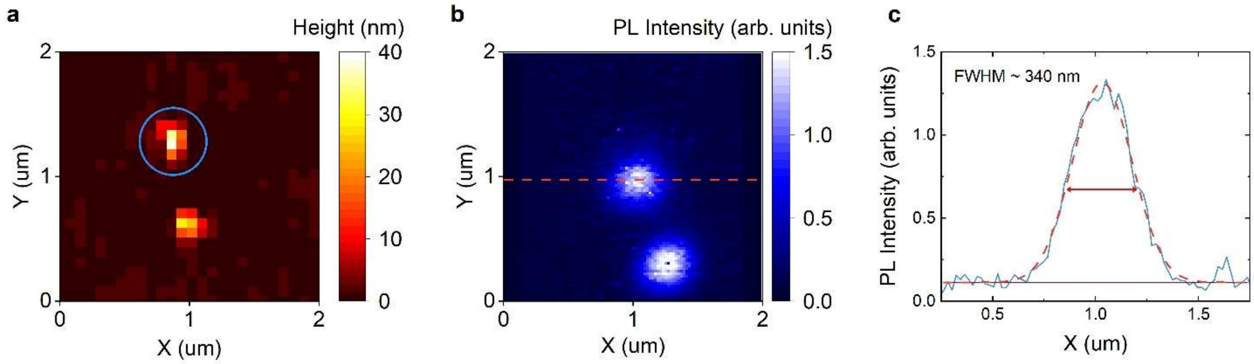

Figure S2. (a) AFM scan of TiN NPs, (b) PL Intensity map of the NPs shown in (a); the red dashed line indicates the cross section taken for fitting, (c) Intensity profile from the cross section shown in (b).

In the main text, we treat the PSF of the microscope as a Gaussian distribution with a FWHM equal to the diffraction limit of the excitation laser spot. We show the validity of this assumption by measuring the PL intensity distribution produced by titanium nitride (TiN) nanoparticles (NPs) with diameters ranging from 20 nm to 40 nm. We first confirm that TiN NPs have sizes well below the optical diffraction limit (Figure S2a). Taking PL intensity maps of the same NPs measured in the AFM (Figure S2b), we find that the produced intensity profile fits well to a Gaussian curve with a FWHM of 340 nm (Figure S2c). This is consistent with the result in the main text where the intensity profile of a single NV center in a nanodiamond was found to be Gaussian with a 310-nm FWHM.

3. Theoretical model of the ML-assisted antibunching super-resolution microscopy. To be able to conduct a more detailed study of the ML-assisted approach we trained the ML model on the theoretical data that emulates HBT measurements and studied the performance of the pre-trained regression ML model in the case of two nano-diamonds configured with different geometrical and emission parameters.

Monte-Carlo simulation of the autocorrelation experiment. In the study, the NV center was represented as a three-level system consisting of an excited state (E), a ground state (G), and a metastable state (M). The transition rates between these levels were fixed as $\gamma_{\mathrm{GE}} = \gamma_{\mathrm{EG}}$, γ_{EM}, and γ_{MG} respectively. The radiative rate is taken to be similar to that in previously studied NV centers in nanodiamonds[1], while the non-radiative constants that are not affected by the photonic environment were taken from another study[2]. The emission behavior was determined by the time-dependent probability given:

$$P_n = Rr_{bg} + R\left(1 - r_{bg}\right)\left[1 - (1+a)\,exp\left(-\left|n - n_0 + \frac{1}{2}\right|\delta t \lambda_1\right)\right.$$
$$\left. + a\,exp\left(-\left|n - n_0 + \frac{1}{2}\right|\delta t \lambda_2\right)\right], \text{for } n \neq n_0$$

$$P_{n_0} = Rr_{bg} + R\left(1 - r_{bg}\right)\left[1 - (1+a)\,exp\left(-\tfrac{1}{4}\delta t \lambda_1\right) + a\,exp\left(-\tfrac{1}{4}\delta t \lambda_2\right)\right], \quad \text{(S.1)}$$

where $\lambda_1 = \gamma_{EG} + \gamma_{GE}$, $\lambda_2 = \gamma_{MG} + \frac{\gamma_{EM}\gamma_{GE}}{\lambda_1}$ and $a = \frac{\lambda_2}{\gamma_{MG}} - 1$. The expression follows directly from the second-order autocorrelation function for a three-level system[2]. Here we assume that the emission was produced partly by a single quantum emitter and partly by photonic background presenting no autocorrelation features. The parameters of the model are summarized in Table S2.

Table S2. Monte-Carlo simulation parameters.

Model Parameter	Interpretation	Value
t_{max}	Maximum absolute delay	400 ns
$\boldsymbol{\delta t}$	Histogram bin width	2.34 ns
N_{bins}	Total number of histogram bins	215
$\boldsymbol{\gamma}_{\mathbf{EG}}$	Radiative excited-state decay rate	20 MHz
$\boldsymbol{\gamma}_{\mathbf{GE}}$	Excitation rate	20 MHz
$\boldsymbol{\gamma}_{\mathbf{EM}}$	Non-radiative shelving rate	10 MHz[3]
$\boldsymbol{\gamma}_{\mathbf{MG}}$	Non-radiative de-shelving rate	7 MHz[3]
$\boldsymbol{r}_{\mathbf{bg}}$	Intensity fraction of the photonic background in the total emission	$1 - \sqrt{1 - g^{(2)}(0)}$

The simulation of the autocorrelation experiment was performed by counting the number of co-detection events from two virtual detectors and grouping them based on the time delay between the two detector clicks. More details of the Monte-Carlo simulation of the autocorrelation experiment can be found in [4].

Synthetic HBT dataset generation and training of CNN model. The CNN regression model was trained on a dataset generated by Monte-Carlo HBT simulations with parameters that span all $g^{(2)}(0)$ emission rate ranges which are typical for NV centers. Specifically, the theoretical model includes two variable parameters: the ground truth value $g^{(2)}(0)$, and the average co-detection counts per bin N. The latter parameter was defined as the total number of co-detection events in the dataset divided by N_{bins}. We generated datasets of $g^{(2)}(0)$ values spanning the interval between 0.1 and 0.9 with a step of 0.04 and N spanning 5 to 55 with a step of 5. One hundred datasets were generated for each combination of $g^{(2)}(0)$ and N, which resulted in 18000 autocorrelation histograms in total. 70% of all the datasets were used for training, and the remaining 30% served to test the performance of the regression model. Here we used the same exact structure of the CNN model as used for experimental data.

Super-resolution imaging process emulation on synthetic data. To be able to analyze the performance of the pre-trained CNN model in the case of the anti-bunching base super-resolution imaging process, we have simulated imaging, autocorrelation histogram collection process, and CNN-based post-processing using a modified Monte-Carlo simulation for two closely spaced nano-diamonds. Imaging is assumed to be done on $2\mu\text{m} \times 2\mu\text{m}$ area in presence of two nano-diamonds with NV centers. The main idea of the approach is to simulate the autocorrelation data collection process within $2\mu\text{m} \times 2\mu\text{m}$ area on the grid mesh with a 10 nm step size. For each pixel of the grid mesh the Monte-Carlo-based $g^{(2)}(t)$ histogram is generated and stored. The generated dataset is used for retrieving the $g^{(2)}(0)$ map using a pre-trained CNN model and corresponding super-resolved image.

Two-emitter case. The ground truth value $g^{(2)}(0)$ spatial distribution is defined as $g^{(2)}(0, x, y) = 2I_1(x, y)I_2(x, y)/(I_1(x, y) + I_2(x, y))^2$. Here $I_{1,2}(x, y)$ are the intensity distributions of the first and second emitter, which are represented by Gaussian distributions with standard deviations defined by the PSF of the optical system. For the self-consistency with the experimental study, the standard deviations σ_0 are set to be equal to 144 nm (PSF = 340 nm FWHM). The resulting intensity distribution of the system of two emitters is defined as $I(x, y) = I_1(x, y) + I_2(x, y)$. Excitation rates for both emitters are set to be proportional to corresponding intensities $k_{exc,j} \sim I_j(x, y), j = 1,2$. The excitation wavelength $\lambda_0$is set to 638 nm, while the NA of the microscope is set to 1.4. Throughout the simulation background noise level is set to be equal to 5% of max($I_1(x, y) + I_2(x, y)$).

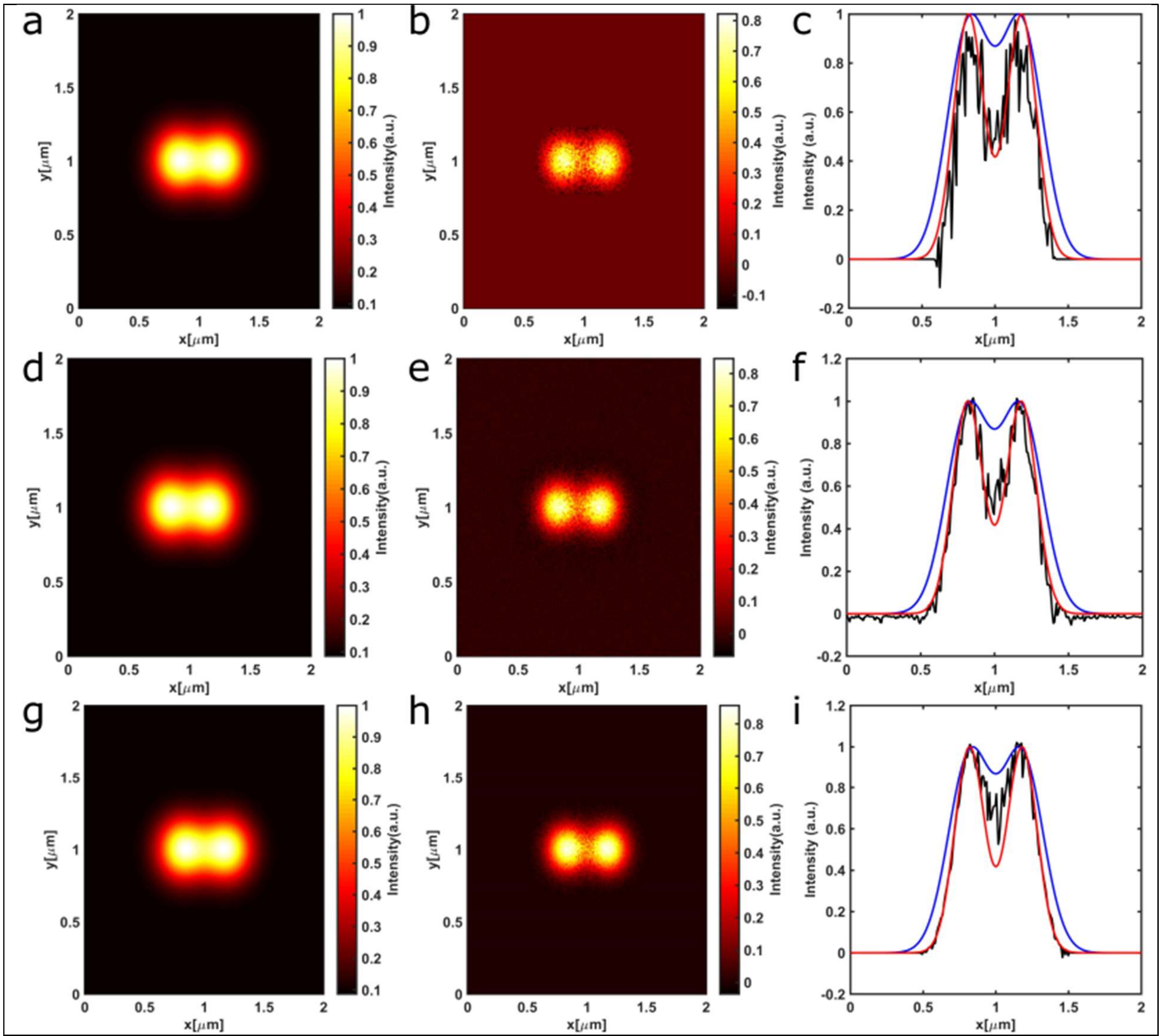

Fig S3. $D = 1.3 \times d_{Rayleigh}$ case. (a) The diffraction-limited intensity distribution of two emitter with $D = 1.3 \times d_{Rayleigh}$ and average counts per bin $N = 5$; (b) ML-assisted super-resolved image; (c) The cross-section of the intensity distribution for diffraction-limited intensity (blue) and super-resolved image (black). The red curve shows distribution of intensity of two emitters with separation $1.3 \times d_{Rayleigh}$ and $\sigma_0/\sqrt{2}$; (d)-(f) the same as (a)-(c) but for $N = 10$; (g)-(i) The same as (a)-(c) but for $N = 20$

Moreover, the Monte-Carlo model has been modified to consider the presence of the second emitter in the vicinity of the first emitter along with the background noise. This is done by

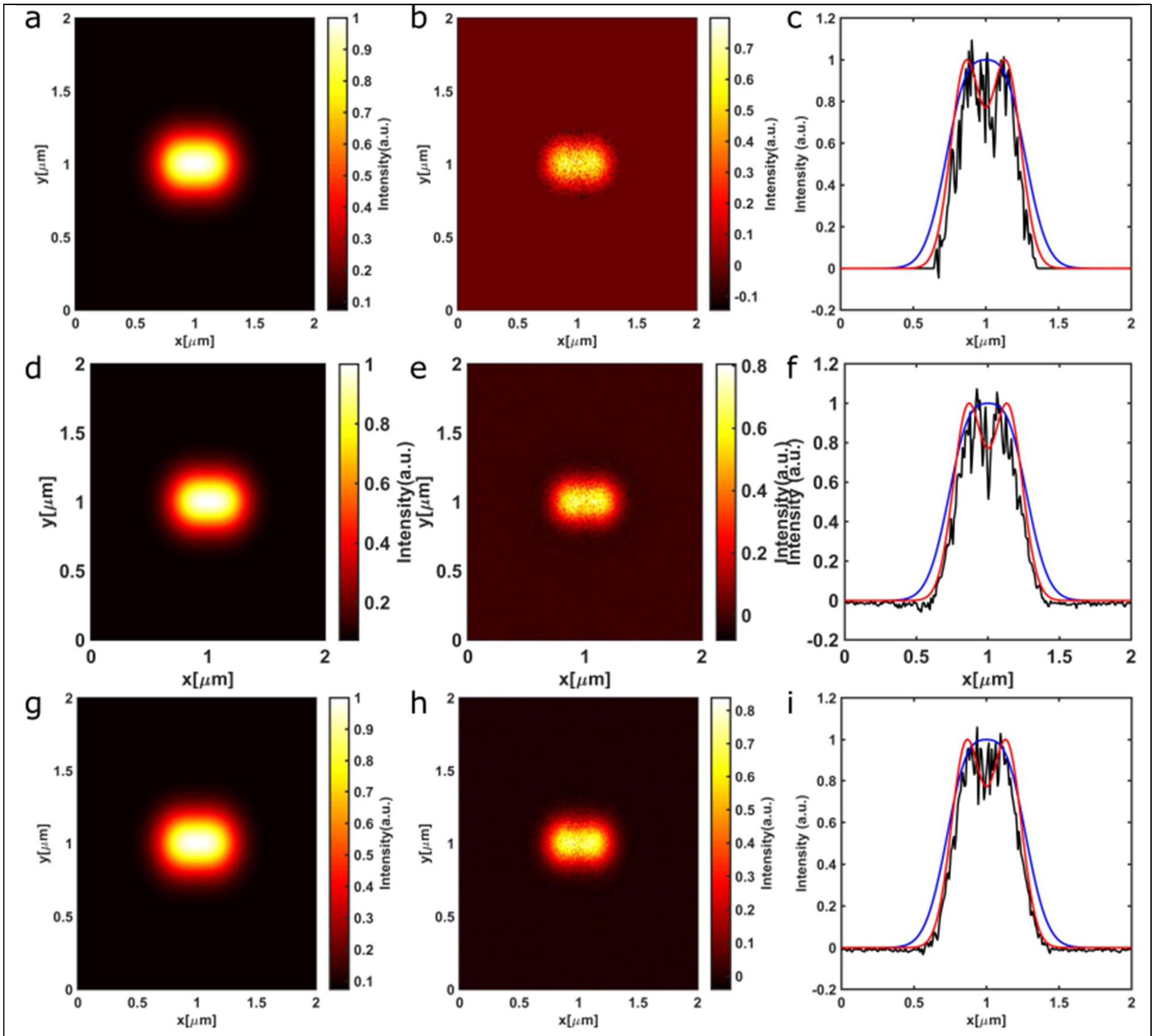

Fig S4. $D = d_{Rayleigh}$ case. (a) The diffraction-limited intensity distribution of two emitter with $D = d_{Rayleigh}$ and average counts per bin $N = 5$; (b) ML-assisted super-resolved image; (c) cross section of the intensity distribution for diffraction-limited intensity (blue) and super-resolved image (black). The red curve shows distribution of intensity of two emitters with separation $1.3 \times d_{Rayleigh}$ and $\sigma_0/\sqrt{2}$; (d)-(f) the same as (a)-(c) but for $N = 10$; (g)-(i) the same as (a)-(c) but for $N = 20$

modification of the detection probability of photon from i-th emitter such that, it is defined by the ratio of the $I_i(x,y)/\left(I(x,y) + I_{bg}\right)$ at a given pixel. Here I_{bg} represents the normalized intensity of the background noise.

Figures S3 and S4 show the main results of the synthetic model-based ML-assisted super-resolution for two different cases of separation between nano-diamonds $D = 1.3 \times d_{Rayleigh}$ and $D = d_{Rayleigh}$, where $d_{Rayleigh} = 0.61\lambda_0/NA$ stands for the Rayleigh distance. The first column in both figures shows the diffraction-limited intensity distribution $I(x,y)$ of two

emitters. Using a pre-trained CNN model, the special distribution of the $g^{(2)}(0)$ is determined, which has been used for super-resolution image retrieval. The second column shows the super-resolved images for the corresponding two nano-diamond configurations. The last column shows the intensity cross-section for diffraction-limited (blue) and super-resolved (black) images. For the sake of comparison, the red curve shows the "ideal" case of a super-resolved image that corresponds to the imaging of two nano-diamonds with PSF with reduced $\sigma_0/\sqrt{2}$. Figure S3 shows the analysis done for the case of $D = 1.3 \times d_{Rayleigh}$. Figures S3 (a)-(c) show the case of NV centers with average counts per bin $N = 5$, (d)-(f) $N = 10$ and (g)-(i) $N = 20$. Here we can see that in all cases the ML assisted super-resolution approach shows an increase in the resolution by a factor of $\sqrt{2}$ as expected. Reduction of the average counts per bin value in the histograms impacts the performance of the CNN regression model, which results in noisier images. Figure S4 shows the same analysis done for the $D = d_{Rayleigh}$ cases. As in $D = 1.3 \times d_{Rayleigh}$, this analysis shows that ML-assisted anti-bunching microscopy increases resolution.

Three-emitter case. In the case of 3-emitters the ground truth value $g^{(2)}(0)$ spatial distribution is defined as $g^{(2)}(0, x, y) = 1 - \sum_{i=1}^{3} I_i^2/(I_1(x, y) + I_2(x, y) + I_3(x, y))^2$. Here $I_{1,2,3}(x, y)$ are the intensity distributions of the first and second emitter, which are represented via Gaussian distribution with standard deviations defined by the PSF of the optical system. For the self-consistency with the experimental study, the standard deviations σ_0 are set to be equal to 144 nm (PSF = 340 nm FWHM). The resulting intensity distribution of the system of two emitters is defined as $I(x, y) = I_1(x, y) + I_2(x, y) + I_3(x, y)$. As in the case of two emitters the excitation rates for emitters are set to be proportional to corresponding intensities $k_{exc,j} \sim I_j(x, y), j = 1,2,3$. The simulation background noise level is again set to be equal to 5%.

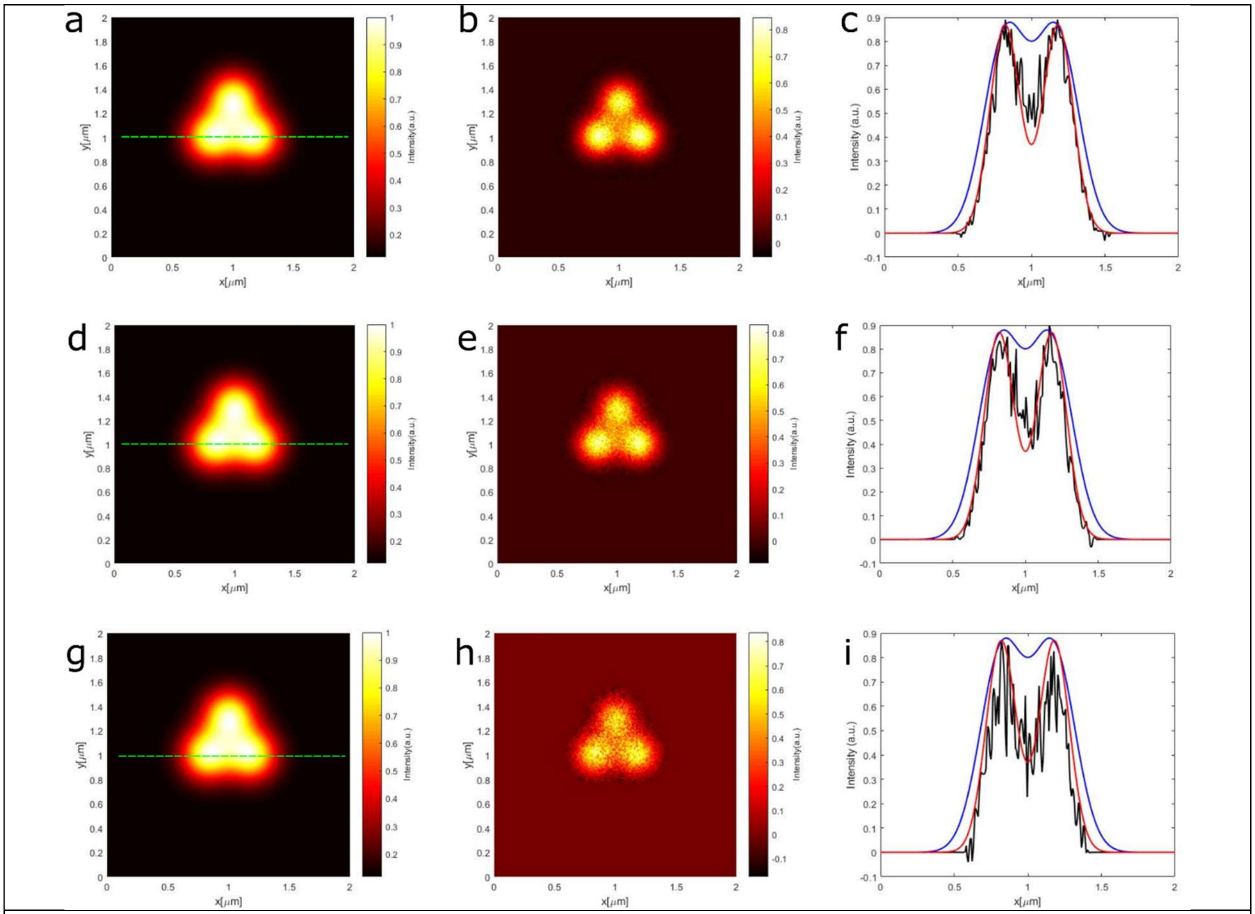

Fig S5. $D = 1.3 \times d_{Rayleigh}$ case for 3 emitters configuration. (a) The diffraction-limited intensity distribution of two emitter with $D = 1.3 \times d_{Rayleigh}$ and average counts per bin $N = 5$ (dashed line shows the cross-section direction); (b) ML-assisted super-resolved image; (c) cross section of the intensity distribution for diffraction-limited intensity (blue) and super-resolved image (black) along dashed line shown in first row images. Red curve shows distribution of intensity of two emitters with separation $1.3 \times d_{Rayleigh}$ and $\sigma_0/\sqrt{2}$; (d)-(f) the same as (a)-(c) but for $N = 10$; (g)-(i) the same as (a)-(c) but for $N = 20$

Figure S5 shows the results of the synthetic model-based ML-assisted super-resolution for three emitters in nanodiamonds separated by $D = 1.3 \times d_{Rayleigh}$. As in previous figures, the first column shows the diffraction-limited intensity distribution $I(x, y)$ of two emitters, while the second column shows the super-resolved images for the corresponding two nano-diamond configurations. The last column shows the intensity cross-section for diffraction-limited (blue) and super-resolved (black) images, and the red curve shows the "ideal" case of a super-resolved image that corresponds to the imaging of two nano-diamonds with PSF with reduced $\sigma_0/\sqrt{2}$. Here we again see that in all cases of average counts per bin values, the ML assisted super-resolution approach shows an increase in the resolution by the expected factor of $\sqrt{2}$.

Supplementary References

1. Bogdanov, S. I. et al. Ultrabright Room-Temperature Sub-Nanosecond Emission from Single Nitrogen-Vacancy Centers Coupled to Nanopatch Antennas. Nano Lett. 18, 4837–4844 (2018).
2. Gupta, A., Hacquebard, L. & Childress, L. Efficient signal processing for time-resolved fluorescence detection of nitrogen-vacancy spins in diamond. J. Opt. Soc. Am. B 33, B28 (2016).
3. Kitson, S. C., Jonsson, P., Rarity, J. G. & Tapster, P. R. Intensity fluctuation spectroscopy of small numbers of dye molecules in a microcavity. Phys. Rev. A 58, 620–627 (1998).
4. Kudyshev, Z.A., Bogdanov, S.I., Isacsson, T., Kildishev, A.V., Boltasseva, A. and Shalaev, V.M. (2020), Rapid Classification of Quantum Sources Enabled by Machine Learning. Adv. Quantum Technol., 3: 2000067 (2020). https://doi.org/10.1002/qute.202000067
